# The Effect of Risk Perception on the 2009 H1N1 Pandemic Influenza Dynamics

**DOI:** 10.1371/journal.pone.0016460

**Published:** 2011-02-07

**Authors:** Piero Poletti, Marco Ajelli, Stefano Merler

**Affiliations:** 1 Bruno Kessler Foundation, Trento, Italy; 2 Department of Mathematics, University of Trento, Trento, Italy; University of Hong Kong, Hong Kong

## Abstract

**Background:**

The 2009 H1N1 pandemic influenza dynamics in Italy was characterized by a notable pattern: as it emerged from the analysis of influenza-like illness data, after an initial period (September–mid-October 2009) characterized by a slow exponential increase in the weekly incidence, a sudden and sharp increase of the growth rate was observed by mid-October. The aim here is to understand whether spontaneous behavioral changes in the population could be responsible for such a pattern of epidemic spread.

**Methodology/Principal Findings:**

In order to face this issue, a mathematical model of influenza transmission, accounting for spontaneous behavioral changes driven by cost/benefit considerations on the perceived risk of infection, is proposed and validated against empirical epidemiological data. The performed investigation revealed that an initial overestimation of the risk of infection in the general population, possibly induced by the high concern for the emergence of a new influenza pandemic, results in a pattern of spread compliant with the observed one. This finding is also supported by the analysis of antiviral drugs purchase over the epidemic period. Moreover, by assuming a generation time of 2.5 days, the initially diffuse misperception of the risk of infection led to a relatively low value of the reproductive number 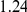, which increased to 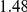 in the subsequent phase of the pandemic.

**Conclusions/Significance:**

This study highlights that spontaneous behavioral changes in the population, not accounted by the large majority of influenza transmission models, can not be neglected to correctly inform public health decisions. In fact, individual choices can drastically affect the epidemic spread, by altering timing, dynamics and overall number of cases.

## Introduction

Among the many factors known to influence the spread of epidemics across human populations, a central role is played by the characteristics of the pathogen responsible for the infections [1], [2], human mobility patterns [3]–[8], the sociodemographic structure of the population [8], [9] and intervention measures [1], [10]. Changes in human behaviors are largely suspected to play a crucial role as well [11]–[15]. As mathematical modeling becomes a powerful tool for decision making both in pre-planning [16]–[25] and in real-time situations [26]–[30], knowing in advance how to account for spontaneous behavioral changes would greatly improve the predictive power of epidemic transmission models and the evaluation of the effectiveness of control strategies.

In March 2009 a new influenza virus emerged in Mexico [31]. Early in the course of the pandemic the population was very concerned about the event [32], [33]. Did this affect the behavior of the population and, consequently, alter the dynamics of the epidemic? By analyzing the 2009–2010 Influenza-Like Illness (ILI) incidence in Italy, as reported to the national surveillance system, the hypothesis appears plausible that spontaneous behavioral changes have played a role in the pandemic, contributing to change the timing of spread and the transmissibility potential. In fact, after an initial period (September–mid-October 2009) characterized by a slow exponential increase in the weekly ILI incidence, a sudden and sharp increase of the growth rate was observed by mid-October. Over the whole period schools remained open [34] and only moderate mitigation measures were enacted (e.g., antiviral treatment of severe cases) [35]. However, during the initial phases of the epidemic the Italian population has been exposed to a massive information campaign on the risks of an emerging influenza pandemic, which can have contributed to alter the perceived risk. The aim of this study is to investigate the effects of the perceived risk of infection during the course of the 2009 H1N1 pandemic in Italy.

Here, we propose a new modeling framework accounting explicitly for the dynamics of behavioral patterns adopted across the population. The idea is that human behavior is mainly driven by the evaluation of prospective outcomes deriving from alternative decisions and cost-benefit considerations. In such a context, evolutionary game theory represents a rich and natural framework for modeling human behavioral changes [36]–[38]. Specifically, different behaviors adopted by individuals are modeled as different strategies whose convenience is defined by the balance between their payoff functions. In evolutionary game theory, due to the dynamic nature of the mechanisms of evolution, repeated strategic interactions and changes in the payoff functions provide insights into adaptive behaviors [37]–[40]. In epidemic modeling, this results in explicitly considering the infection dynamics as the interplay between the disease transmission process and the spontaneous response of the population, where changes in human behavior (and in particular in the payoff functions) are triggered by the epidemic dynamics and vice versa.

## Materials and Methods

### Data description

In Italy, influenza surveillance system INFLUNET (accessible at: http://www.iss.it/iflu/) is based on a nationwide voluntary sentinel network of general practitioners and pediatricians. The aim is to monitor ILI incidence and to collect information on circulating strains. Incidence rates are based on the population served by each reporting physician each week.

As most European countries, Italy has experienced one single pandemic wave during fall-winter 2009 and no substantial activity has been detected during the summer [30]. The pandemic has mainly spread starting since the reopening of schools in mid-September until mid-December. Over this period, only mild mitigation strategies, including treatment of the most severe cases with antiviral drugs [35] and a moderate vaccination program, were performed. More in detail, the vaccination program started on mid-October and involved a small fraction of at-risk patients and essential workers (over the whole course of the pandemic less than 1.5% of the Italian population was vaccinated [35]). Finally, in the considered period, schools remained open as regular holidays were not scheduled [34].

We consider total ILI incidence from week 38 (corresponding to the reopening of schools after the summer break, when influenza activity started to be detected by the surveillance system) to 50, 2009. This allows us to investigate an “uncontrolled” epidemic, not affected by heavy public health interventions or by school closure. The number of practitioners involved in the surveillance system over the considered period varies from 561 to 1,165; consequently, the served population varies from 767,154 and 1,509,971. These values guarantee the reliability of the number of weekly reported cases.

### The model

The transmission process is based on a Susceptible-Infective-Recovered (SIR) model where susceptible individuals may adopt two mutually exclusive behaviors, “*normal*” and “*altered*”, on the basis of the perceived risk of infection. Individuals adopting *altered* behavior are supposed to be able to reduce the risk of infection by reducing the force of infection to which they are exposed. This reduction can be achieved by reducing the number (or the type) of physical contacts or by adopting self-prophylactic measures aimed to reduce the transmission probability during contacts. For instance, a self reduction in the number of contacts can occur through the avoidance of crowded environments or by limiting travels. On the other hand, a reduction in transmission probability can be achieved by washing hands frequently or following cough/respiratory etiquette, as recommended by the WHO [41].

In the model, individuals can change their behavior spontaneously, on the basis of cost/benefit considerations. This phenomenon perfectly fits to the language of evolutionary game theory, in which behaviors adopted by individuals correspond to strategies played in a suitable game, with certain expected payoffs: the *altered* behavior takes the advantage of reducing the risk of infection, but it is more costly (e.g., because individuals have to limit their activities). Which behavior is more convenient to adopt clearly depends on the state of the epidemic. The balance of the payoff between the two possible behaviors is determined by the perceived risk of infection, which depends on the cost associated to the risk of infection and on the perceived prevalence of infections in the population. The latter is modeled by assuming a fading memory mechanism (e.g., as in [42], [43]) altering the perception of the risk of infection on the basis of the number of cases occurred over a certain (past) period of time. The diffusion of strategies in the population is modeled as an imitation process [38], [44] based on the idea that individuals change strategy as they become aware that their payoff can increase by adopting another behavior. Denoting by 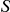, 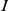, 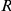 the fraction of susceptible, infective and recovered individuals respectively and by introducing the variables 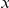, describing the fraction of individuals adopting the *normal* behavior, and 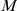, describing the perceived prevalence of infection in the population, the system of ordinary differential equations regulating this process can be written as follows:
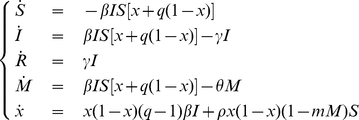
where 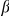 is the transmission rate; 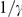 is the average duration of infectivity period (corresponding to the generation time); 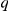 represents the reduction of the risk of infection to which individuals adopting *altered* behavior are exposed; 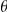 weighs the decay of the perceived prevalence; 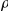 essentially represents the speed of the imitation process with respect to the pathogen transmission dynamics; 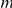 defines the risk threshold for determining which behavior would represent the most convenient choice.

Briefly, the last equation of the system models the diffusion of the two different behaviors in the population driven by an imitation/natural selection process. The first term of the equation accounts for a natural selection embedded into the transmission process that favors individuals reducing the risk of infection, while the second one accounts for an imitation process modeling spontaneous changes in individual behaviors. These changes occur on the basis of the difference between the payoffs of the two possible behaviors, the perceived prevalence, the level of the risk threshold and the speed of the imitation process which, in general, is different from the speed of the disease transmission process (as imitation is based on the diffusion of information rather than on physical contacts between individuals). Model details are presented in Text S1.

The basic reproductive number 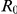, which is essentially the average number of secondary infections that results from a single infectious individual in a fully susceptible population [1], can be computed by using next generation technique [45]. The resulting basic reproductive number is 
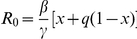
, which can be interpreted as a weighted sum of two basic reproductive numbers: 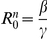, the reproductive number for individuals adopting the *normal* behavior (namely the fraction 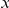) and 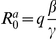, the reproductive number for individuals adopting the *altered* behavior (namely the fraction 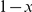). Therefore, 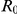 depends on the fraction of individuals in the population who are currently adopting either *normal* or *altered* behavior.

### Model calibration

In order to capture the factors underlying the spread of the 2009 H1N1 pandemic, we fit the proposed model to ILI incidence data by a least-squares procedure. Specifically, the weekly incidence 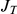 predicted by model simulations can be computed as 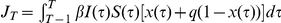. Thus, for a given choice of model parameters and of the reporting factor 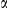, the square error 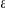 between predicted and observed incidence can be estimated as 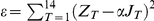, where 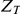 represents the weekly ILI incidence as reported to the Italian surveillance system on week 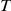 (week 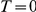 corresponds to week 37, 2009). The optimization procedure is a random walk stochastic local search algorithm [46].

According to a serological survey on the Italian population, the fraction of naturally immune individuals before the beginning of the pandemic was about 10% [47]. Therefore, the initial fraction of susceptible individuals in the model was set to 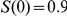. The generation time is assumed 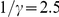 days [48]–[50]. Moreover, the initial fraction of individuals adopting the *altered* behavior is assumed to be 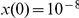 (i.e., almost the whole population is initially adopting the *normal* behavior). Finally, given the interdependence between 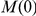 and 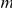 (see the discussion in Text S1), the value of 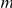 is kept fixed to 0.1. All other parameters (namely, 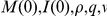, 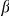 and 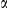) are estimated through the least-squares fit. A detailed sensitivity analysis of the model is presented in Text S1.

Two additional models are included in the performed analysis: the “classical” SIR model and a SIR model assuming time-dependent transmission rate, which is considered to be a step function switching between two values at a given time. The initial fraction of susceptible individuals and the generation time are kept fixed (as in the model accounting for behavioral changes), while all other parameters are estimated via model fit. Specifically, for the SIR model the fitted parameters are the reporting factor, the initial fraction of infective individuals and the transmission rate; for the SIR model with time-dependent transmission rate the fitted parameters are the reporting factor, the initial fraction of infective individuals, two transmission rates and the time at which the switch between these two values occurs.

## Results

**Figure 1:**
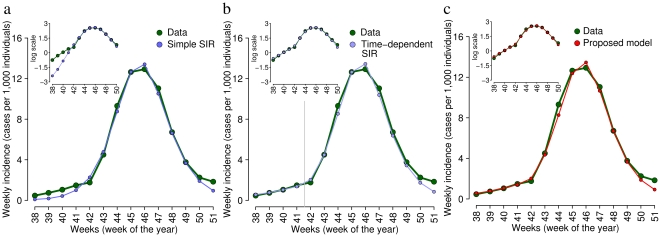
Comparing observed ILI incidence and model simulations. **a** Weekly ILI incidence as reported to the surveillance system (green) and weekly incidence simulated by a “simple” SIR model (blue). Sub-panel shows the same curves in a logarithmic scale. Parameter values assumed in the simulation are: the generation time 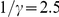 days [48]–[50] and 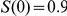, according to a serological survey on the Italian population [47]. Parameter values estimated via model fit are: 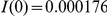, 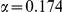 and 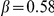. **b** Weekly ILI incidence as reported to the surveillance system (green) and weekly incidence simulated by a SIR model assuming a time-dependent transmission rate (blue). Sub-panel shows the same curves in a logarithmic scale. Assumed parameters are: 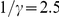 days and 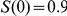. Values of the fitted parameters are: 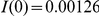, 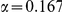, 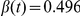 for weeks 38–41.58 and 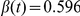 for weeks 41.58–51. **c** Weekly ILI incidence as reported to the surveillance system (green) and weekly incidence simulated by the proposed model (red). Sub-panel shows the same curves in a logarithmic scale. Assumed parameters are: 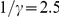 days, 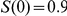, 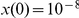 and 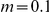. The values of the fitted parameters are: 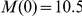, 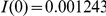, 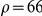, 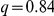, 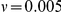, 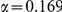 and 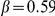. In addition, the estimates of the reporting factor 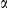 as obtained by fitting the three models and reported in a, b and c (namely, 17.4%, 16.7% and 16.9%, respectively) are in good agreement with the range 18%–20.2% estimated in [30].

The ILI incidence as reported to the surveillance system during the 2009 H1N1 pandemic shows two different phases characterized by two distinct exponential growth rates, especially appreciable when data are plotted in a logarithmic scale (see Fig. 1a and its sub-panel). The “classical” SIR model is not able to catch this phenomenon (see Fig. 1a) unless considering a time-dependent transmission rate, switching from a low transmission level during the first four weeks to a higher level for the rest of the epidemic (see Fig. 1b). However, this model is not able to explain the motivation underlying this sudden change in the transmissibility potential.

On the contrary, the model introduced here perfectly fits the observed ILI incidence (see Fig. 1c) providing a plausible explanation of the mechanisms responsible for the observed pattern. Specifically, the estimated parameter configuration obtained by fitting ILI incidence entails an initial overestimation of the perceived risk that decreases over time, along with an initial diffusion of the *altered* behavior in the population which becomes replaced by the *normal* behavior during the course of the epidemic. In fact, a high level of perceived risk of infection at the beginning of the pandemic leads the simulated population to adopt the *altered* behavior (as in the presence of a well sustained circulation of the virus) resulting in a growth rate of the epidemic lower than what would have been observed in a population adopting the *normal* behavior. Along with a slow increase in the number of cases, a decrease in the perceived risk of infection is observed. In fact, the latter depends on the combination of these two opposite phenomena: the increase of new infections and the decline of the perceived prevalence (slowed by the memory mechanism), which was overestimated in the early phases of the epidemic. As the perceived prevalence goes below the risk threshold 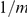, the *normal* behavior starts to spread quickly in the population as the most convenient strategy to be adopted through the subsequent course of the epidemic. This induces an increase of 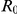 which leads to a sudden change in the growth rate of the epidemic. Thus, these two distinct exponential growth phases in the observed ILI incidence correspond to two phases in the model: the first one characterized by the diffusion of the *altered* behavior in the population and thus driven by 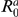, and the second one characterized by the diffusion of the *normal* behavior and thus driven by 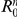. The best estimate for 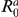 is 1.24 and for 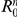 is 1.48 (which correspond to two effective reproductive numbers of 1.12 and 1.33 respectively, given a 10% initial natural immunity to the 2009 H1N1pdm strain in the Italian population [47]). The basic reproductive number estimated in the phase of the epidemic characterized by the *normal* behavior, namely 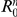, is in good agreement with the estimates previously obtained for the 2009 pandemic in Italy [30] and in several regions of the world [28], [48]–[51].

The analysis of the sensibility of the model to changes in parameter values highlights that the model complies with observed ILI incidence only if an initial (persistent) diffusion of the *altered* behavior in the population is considered (see Text S1). Specifically, an initial perceived risk of infection above the risk threshold, a long-lasting memory mechanism (able to maintain the *altered* behavior as more convenient over a relevant period of time) and a fast imitation process (enough to produce a sudden change in the force of infection) are required. Moreover, model predictions are robust in terms of final epidemic size (with absolute differences of the order of 3%), while they are more sensible in terms of timing of the epidemic. Specifically, small variations in the reduction of the risk of infection in individuals adopting the *altered* behavior result in changing 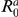 and thus the timing of the epidemic. The same holds if the initial perceived prevalence and the risk threshold determining which behavior is more convenient to adopt are perturbed. On the other hand, no relevant differences appear by increasing either the average duration of the perceived risk of infection (i.e., the length of the long-lasting memory mechanism) or the speed of the imitation process. A further analysis (shown in Text S1) has revealed that, if the risk of infection is overestimated during the early phases of the epidemic, the diffusion of the virus would be slowed down (thus gaining time for vaccine production). On the other hand, if such overestimation occurs during the outbreak, a lower peak incidence (and thus a lower burden for health care centers) and a relevant decline of the final epidemic size would be observed.

Results reported above support the hypothesis that the mass media campaign on the risks of an emerging influenza pandemic performed in the early phases of the epidemic might have induced a high perceived risk of infection at the beginning of the pandemic, as it has been highlighted by specific survey studies [32], [33]. In order to investigate if such phenomenon could have been a peculiarity of the 2009 pandemic, we also analyzed the last three influenza seasons in Italy. Our analysis, reported in Text S1, reveals that during the 2006–2007, 2007–2008 and 2008–2009 influenza seasons behavioral changes would not have played a relevant role in the early phases of the epidemics.

**Figure 2:**
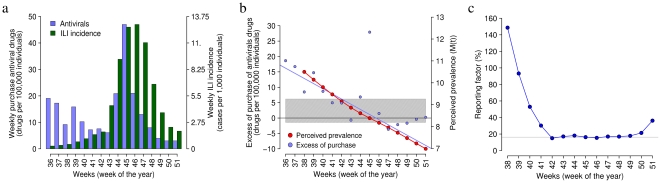
Risk perception, antivirals purchase and reporting factor. **a** Weekly purchase of antiviral drugs (light blue, scale on the left axis) and weekly ILI incidence as reported to the surveillance system (green, scale on the right axis) during the 2009–2010 pandemic in Italy. **b** Light blue points (scale on the left axis) represent the weekly excess of the purchase of antiviral drugs. The latter is defined as the difference between the actual and the expected amount of antiviral drugs purchased (which is assumed to be proportional to ILI incidence, and the proportionality constant is computed as the number of antivirals purchased divided by the ILI incidence averaged over weeks 43–51, i.e. in the period of sustained transmission). Light blue line represents the best linear model fit to the excess of purchased antivirals. Horizontal black line represents the threshold over which the number of antivirals purchased is larger than the expected one. Grey area represents the maximum and the minimum excess of antiviral drugs purchased over the weeks 43–51. Red points (scale on the right axis) represent the perceived prevalence of infection simulated by the model parameterized as in Fig. 1c. **c** Weekly reporting factor estimates that enable the simple SIR model (parameters as in Fig. 1a) to exactly fit the reported ILI incidence. The horizontal gray line represents the average reporting factor as computed over the weeks 42–50.

Beyond the previous analysis, the hypothesis of an overestimation of the risk is supported by the temporal pattern of drug purchases and by sporadic self-imposed school closures. Specifically, during the 2008–2009 influenza season the maximum weekly number of antiviral drugs sold was under 2 doses per 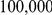 individuals per week, while when the 2009 pandemic arrived in Europe (end of April) antiviral drug purchases immediately jumped to more than 12 doses per 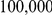 individuals per week [52]. Despite no substantial ILI activity has been detected in Italy during the summer, the purchase of antiviral drugs reached a peak of about 35 doses per 100,000 individuals per week at the end of July. As shown in Fig. 2a and 2b, during fall the purchase of antiviral drugs complies with the observed ILI temporal dynamics, while until mid-October an excess of antiviral drug purchase can be observed, supporting the hypothesis of an initial overestimation of the risk of infection. On the contrary, the sales of pain killers (which are commonly used to relieve pain due to mild symptoms) have followed a completely different pattern: during the summer the sales have been (nearly) constant, then they started to increase from the middle of September [52]. The purchase of antiviral drugs might have been amplified by the concern about the pandemic possibly thanks to the information campaign about the use of antivirals for treating H1N1 infections. Moreover, from the end of September to the beginning of October, a few examples of reactive school closure have been documented [53]–[56]. Such school closures were “self-imposed” by the scholastic board or suggested by local authorities (at municipality level). However, these sporadic closures can hardly be thought as the only responsible for the low transmission observed during the early phases of the epidemic. These two examples, however, provide empirical evidence that a high risk for the ongoing pandemic influenza has been perceived by the Italian population and that individuals have actively performed spontaneous defensive response measures aimed to reduce the risk of infection.

Behavioral changes, though not directly affecting the transmission process, can also be relevant from an epidemiological perspective. For example, as well known by epidemiologists, a high perceived risk during an epidemic can increase the notification rate, especially if the surveillance system is based on consultations. However, such phenomenon does not seem to be able to explain (alone) the observed pattern. In fact, a simple SIR model, with a time-dependent reporting factor, can capture ILI incidence during the initial phase of H1N1 pandemic only by considering extremely large values of the reporting factor (even above 100%, see Fig. 2c). Similar results have been obtained by investigating a dataset combining results from the virological and epidemiological surveillance systems which allowed the estimation of a theoretic lower bound of the number of H1N1pdm infections in Italy (see Text S1).

Finally, in order to increase model realism, a latent class of individuals can be added to the proposed model. However, as shown in Text S1, qualitative results do not substantially change: model fit is still in excellent agreement with ILI incidence data. This result confirms that the essential dynamics can be captured by considering the SIR-version of the proposed model since the latent period of influenza is quite short. Nonetheless, in other contexts, the inclusion of a latent class may remarkably affect the transmission dynamics.

**Figure 3:**
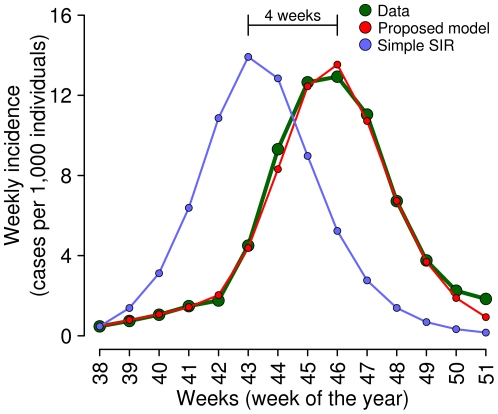
The impact of risk perception. Weekly ILI incidence as reported to the surveillance system (green) and incidence simulated by the model (red; parameter values as in Fig. 1c). Weekly incidence simulated by the “classical” SIR model (blue; parameter values as in Fig. 1a but for 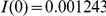).

As a matter of fact, if changes in human behavioral patterns (such as self-protection) are not taken into account, two opposite outcomes can be observed. Firstly, estimates of the growth rate based on the observations during the early phases of the epidemic may lead to an underestimation of the transmissibility potential of the disease and thus to underrate the impact of the epidemic. Secondly, predictions based on robust available estimates of the reproductive number (e.g., taken from the analysis in countries where the epidemic is already well sustained) would lead to overestimate the growth rate of the epidemic during its early phases, resulting in turn in predicting a faster spread than the actual one. For instance, by using a SIR model, accounting for the best parameter estimates as obtained by fitting the entire epidemic but initializing the system with the actual number of cases at the beginning of autumn 2009 (namely on week 38), the simulations reach the epidemic peak four weeks in advance with respect to the actual pandemic (see Fig. 3). A similar result has been observed also in [30], where - using a model not accounting for behavioral changes in the population - the epidemic peak has been predicted two weeks in advance with respect to the actual value.

## Discussion

A high level of the perceived risk at the beginning of the 2009–10 pandemic is largely suspected, as well as its effect in slowing the epidemic spread. Our aim here is to validate this hypothesis by showing that spontaneous behavioral changes in the population might have played a central role in the early phases of the pandemic in Italy. Specifically, ILI incidence shows a low (though exponential) growth, followed by a sudden change in its growth rate starting from week 42. Such pattern can hardly be captured by classical models. On the contrary, the proposed approach is able to reproduce it by explicitly modeling spontaneous behavioral changes in the population. Our analysis is supported by some empirical evidence (e.g., the purchase of antiviral drugs) and reveals that a high perceived initial risk of infection could be a plausible explanation for such phenomenon.

This study represents a first step for the estimation of the quantitative and qualitative effects of spontaneous behavioral changes in the population on the spread of epidemics. We believe it provides a promising approach, based on evolutionary game theory, for including the behavior dynamics into epidemic transmission models. The proposed approach is general enough to be used for describing any kind of disease where spontaneous behavioral changes could play a relevant role. Similar approaches have been previously used to investigate individual choices in non-compulsory vaccination programs [42], [44], [57]–[59].

As shown in this study, behavioral changes (e.g. induced by mass media information campaigns) can significantly affect the epidemic spread both qualitatively (e.g., by altering the epidemic dynamics) and quantitatively (e.g., by substantially slowing the epidemic spread or by determining different final epidemic sizes). Therefore, considering an approach accounting for spontaneous behavioral changes would be helpful for giving insight to public health policy makers, for planning public health control strategies (e.g., vaccination) and better estimating the burden for health care centers over time. Moreover, this study highlights that the estimation of fundamental epidemiological parameters (and in particular the reproductive number) could be largely affected by human behaviors.

At the current stage, the proposed model could hardly be used for real time predictions since our knowledge on plausible values of model parameters related to human behavior is only preliminary. Further investigations on the 2009–2010 pandemic dynamics in other countries or on other epidemics where behavioral changes have been suspected (e.g., the 2002–03 SARS outbreak) have to be performed in order to gain a major consciousness on how such mechanisms work.

## Supporting Information

Text S1 **In this appendix model derivation and its SEIR variant are presented along with an in-depth sensitivity analysis.** Moreover, an investigation of past influenza seasons and of virological surveillance data is carried out.”
(PDF)
