# Supplementary material for: The Effect of Risk Perception on the 2009 H1N1 Pandemic Influenza Dynamics
Source: PLoS One. 2011 Feb 7;6(2):e16460. doi: 10.1371/journal.pone.0016460 (PMC3034726; doi:10.1371/journal.pone.0016460)
Supplement: Text S1 — In this appendix model derivation and its SEIR variant are presented along with an in-depth sensitivity analysis. Moreover, an investigation of past influenza seasons and of virological surveillance data is carried out.” (PDF) [file pone.0016460.s001.pdf]

The effect of risk perception on the 2009 H1N1 pandemic influenza dynamics

Piero Poletti[1,2], Marco Ajelli[1] & Stefano Merler[1]

[1] Bruno Kessler Foundation, Trento, Italy,
[2] Department of Mathematics, University of Trento, Italy

Supporting Text S1

Contents

Contents 1

1 The model 2

2 Including a latent class of individuals 4

3 Sensitivity analysis 5

4 Alert time 10

5 Past influenza seasons 11

6 Combining epidemiological and virological surveillance data 13

Bibliography 15

1 The model

The aim here is to introduce a model for a disease transmission process, accounting for heterogeneity with respect to the behaviors adopted spontaneously by the individuals of the host population. The diffusion of the different behaviors performed by the population is modeled through evolutionary game theory. Specifically, we introduce a simple SIR model where individuals can adopt two mutually exclusive behaviors on the basis of the perceived risk of infection through a classical imitation process. We decided to keep the transmission model as simple as possible. Therefore no latency period, age classes, different levels of symptomaticity, variable viral load over time are considered. The host population is assumed to be divided into three classes, namely susceptible (S), infective (I) and recovered (R) individuals.

We assume that individuals are able to reduce the force of infection to which they are exposed as a spontaneous defensive response to the epidemic. Actually, individuals exposed to the risk of infection are only susceptible ones. As we are considering the dynamics of a spontaneous self–protection strategy that reduces the undergoing force of infection, neither infective, nor recovered individuals can achieve any benefit through a reduction of their force of infection, unless by assuming an altruistic interaction between them and susceptible individuals. Since such investigation is beyond the scope of this work, we consider only behavioral changes among susceptible individuals. Therefore, susceptible individuals are divided in two subclasses: individuals adopting a *"normal"* behavior (S_n) and individuals adopting an *"altered"* one (S_a), assuming that the latter are able to reduce the received force of infection. From now on, let us denote as b_n and b_a the two different behaviors adopted by S_n and S_a respectively.

Let us define β as the transmission rate, $1/\gamma$ the average duration of the infectivity period and $q \in (0, 1)$ the reduction of the force of infection performed by S_a (e.g., by avoiding crowded environments or by increasing wariness in usual activities involving contacts with other individuals). The epidemic flows between classes can be described as follows:

$$\begin{cases} \dfrac{dS_n}{dt}(t) &= -\beta I(t)S_n(t) \\ \dfrac{dS_a}{dt}(t) &= -q\beta I(t)S_a(t) \\ \dfrac{dI}{dt}(t) &= \beta I(t)[S_n(t) + qS_a(t)] - \gamma I(t) \\ \dfrac{dR}{dt}(t) &= \gamma I(t). \end{cases} \tag{1}$$

Setting $x = S_n/(S_n + S_a)$, where x represents the fraction of susceptible individuals adopting the *normal* behavior b_n, system (1) can be rewritten as follows

$$\begin{cases} \dfrac{dS}{dt}(t) &=& -\beta\left[x(t)+q(1-x(t))\right]S(t)I(t) \\ \dfrac{dI}{dt}(t) &=& \beta\left[x(t)+q(1-x(t))\right]S(t)I(t)-\gamma I(t) \\ \dfrac{dR}{dt}(t) &=& \gamma I(t) \\ \dfrac{dx}{dt}(t) &=& x(t)(1-x(t))\left[q\beta I(t)-\beta I(t)\right] \ . \end{cases} \tag{2}$$

where, as mentioned above, $S = S_n + S_a$ is the whole fraction of susceptible individuals.

The latter equation of system (1), obtained by deriving $S_n/(S_n + S_a)$, can be read as a "natural" selection process embedded in the transmission dynamics that favors individuals reducing the force of infection.

Let us assume that individuals can also change strategy spontaneously during the course of the epidemic, through cost–benefit considerations that involve the perceived risk of infection.

This phenomenon perfectly fits to the language of evolutionary game theory, in which behaviors correspond to strategies that are adopted or not on the basis of their convenience. More specifically here we assume that behavior diffusion is driven by an imitation dynamics [1, 2, 3, 4]: a fraction of the individuals playing strategy b_n can switch to strategy b_a after having compared the payoffs of the two strategies, namely p_n and p_a, at a rate proportional to their difference $\Delta P = p_n - p_a$, with proportionality constant ω; conversely for the fraction of the individuals playing b_a. As this comparison is based on the diffusion of information and may not involve only physical contacts between individuals, the imitation process and the pathogen transmission can have two different time scales. Thus let us introduce τ as the time unit for spontaneous behavioral changes, and let us assume that $t = \alpha\tau$ with $\alpha \in \mathbb{R}$.

The convenience of two mutually exclusive behaviors is modeled through their corresponding payoff functions. We consider that all individuals pay a cost for the risk of infection, which we assume to depend linearly on the perceived prevalence, $M(\tau)$, and it is higher for b_n than for b_a. Moreover, individuals playing strategy b_a pay an extra fixed cost. Hence, the payoffs associated with b_n and b_a are respectively:

$$\begin{array}{rcl} p_n(\tau) &=& -m_n M(\tau) \\ p_a(\tau) &=& -k - m_a M(\tau) \end{array} \quad ,$$

with $m_n > m_a$. We may think of m_n and m_a as parameters related to the risk of developing symptoms induced by the two different behaviors b_n and b_a, while k represents the cost of any self-imposable prophylactic measure (e.g., less traveling).

The perceived prevalence M is modeled through an exponentially fading memory mechanism (such as in [5]) as follows:

$$\frac{dM}{dt}(t) \;=\; \beta\left[x(t)+q(1-x(t))\right]S(t)I(t)-\theta I(t) \ .$$

where θ weighs the decay of the perceived risk of infection produced by new cases and thus $1/\theta$ can be read as the average duration of the memory of new cases in the perceived prevalence.

By adding the imitation dynamics in the last equation of system (2), the equation for the fraction of susceptible individuals adopting *normal* behavior x becomes

$$\frac{dx}{dt}(t) \;=\; x(t)(1-x(t))\left[q\beta I(t) - \beta I(t)\right] + \tfrac{\omega}{\alpha} x(t)(1-x(t))S\left[k - (m_n - m_a)M(t)\right] \qquad (3)$$

expressed in the time scale of infection transmission process. Equation (3) can be rewritten in the following form:

$$\frac{dx}{dt}(t) \;=\; x(t)(1-x(t))\beta(q-1)I(t) + \rho x(t)(1-x(t))S\left[1 - mM(t)\right] \;.$$

where $\rho = \frac{\omega k}{\alpha}$, $m = (m_n - m_a)/k$.

As a matter of fact, $1/m$ defines the threshold for the perceived prevalence $M(t)$, over which it is more convenient to adopt the *altered* behavior. Moreover ρ essentially represents the speed of the imitation process with respect to the pathogen transmission dynamics. Finally, q tunes the reduction of the force of infection for individuals adopting the *altered* behavior.

One could consider a more general model for behavior dynamics, including the possibility that individuals can also (rarely) change behavior regardless of cost–benefit considerations, as driven by an *irrational exploration.* This possibility can be modeled as a mutation dynamics [1], not favoring any strategy, by considering a mutation rate $\mu << 1$. The resulting equation for x would become

$$\frac{dx}{dt}(t) \;=\; x(t)(1-x(t))\beta(q-1)I(t) + \rho[x(t)(1-x(t))S(1-mM(t)) - \mu x + \mu(1-x)] \;.$$

Mutation dynamics might be considered to gain more realism (details have been largely discussed in [4]). Nonetheless, its effect can be neglected for the purpose of this work.

2 Including a latent class of individuals

In order to account for a latency period in the disease transmission model, a new class of individuals E, representing the latent individuals, is added to the model. Therefore, the SEIR-version of the model accounting for behavioral changes in the population is regulated by the following system of ordinary differential equations:

$$\begin{cases} \dot{S} &= -\beta I S\left[x + q(1-x)\right] \\ \dot{E} &= \beta I S\left[x + q(1-x)\right] - \omega E \\ \dot{I} &= \omega E - \gamma I \\ \dot{R} &= \gamma I \\ \dot{M} &= \omega E - \theta M \\ \dot{x} &= x(1-x)(q-1)\beta I + \rho x(1-x)(1-mM)S \end{cases} \qquad (4)$$

where $1/\omega$ represents the average duration of the latent period, while all other parameters have the same interpretation given in the main text for the SIR-version of the model.

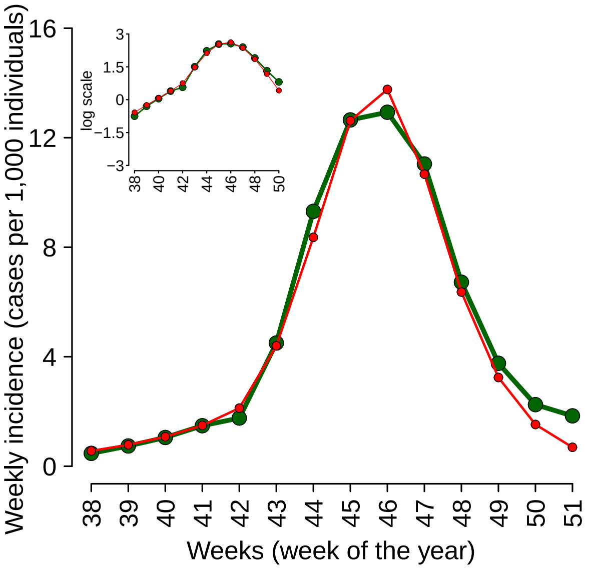

Figure S1: Weekly ILI incidence as reported to the surveillance system (green) and weekly incidence simulated by model (4) (red). Sub-panel shows the same curves in a logarithmic scale. Parameter values assumed in the simulation are: $1/\omega = 0.83$ days, $1/\gamma = 1.67$ days (and thus the resulting generation time is 2.5 days), $S(0) = 0.9$, $x(0) = 10^{-8}$ and $m = 0.1$. The values of the fitted parameters are: $M(0) = 10.5$, $I(0) = 0.001238$, $\rho = 65$, $q = 0.84$, $\nu = 0.005$ and $\beta = 0.9$. The resulting basic reproductive numbers are: $R_0^a = 1.26$ and $R_0^n = 1.5$. The estimated reporting factor is 16.3%.

Model (4) is fitted to the ILI incidence as reported to the surveillance system during the 2009 H1N1 pandemic. As for the analysis performed in the main text, the value of the generation time T_g is assumed 2.5 days. However, in the case of an SEIR model with exponentially distributed infectivity and latent periods, the generation time corresponds to the sum of latent and infectivity periods (i.e., $T_g = 1/\gamma + 1/\omega$). As in [6], the latent period is assumed to be one third of the generation time.

As shown in Fig. S1, even the SEIR-version of the model is able to capture the influenza dynamics. Moreover, the qualitative pattern of the behavior of the population does not change as well. Basically, at the beginning of the epidemic the *altered* behavior is widespread and the perceived prevalence is overestimated (as for the SIR-version, it remains above the threshold until week 39.42). Then, on week 41.67 (week 41.64 in the SIR-version), the individuals adopting the *normal* behavior exceed 50% of the population and the *normal* behavior becomes widespread until the end of the epidemic.

3 Sensitivity analysis

In order to assess the robustness of qualitative results shown in the main text, we investigate the sensibility of the model by changing one–by–one the values of fitted parameters starting from

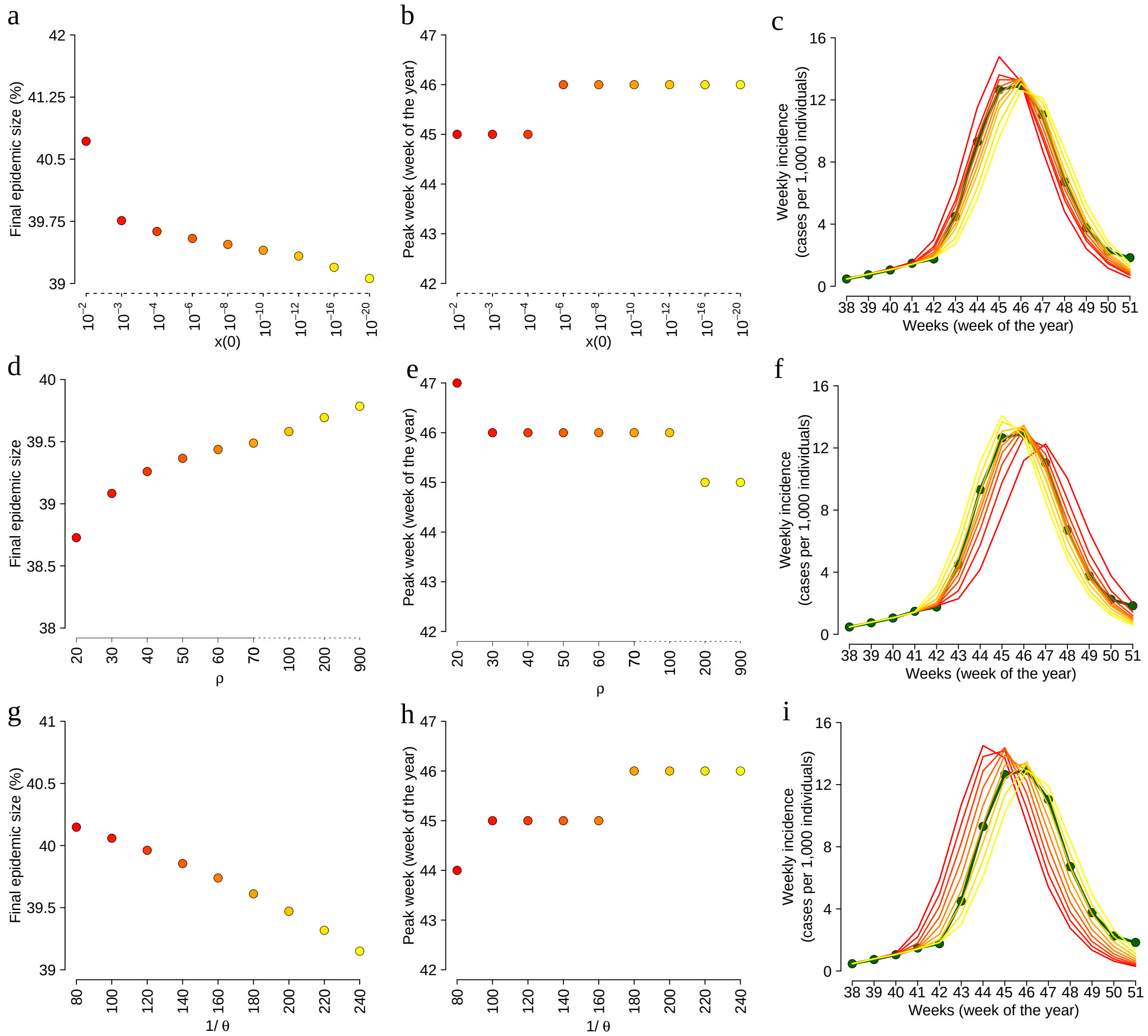

Figure S2: **a** Final epidemic size as simulated by the proposed model for different values of $x(0)$. Other parameters as described in the main text (see Fig. 1). **b** As is **a** but for the peak week. **c** As is **a** but for the weekly incidence. Lines colors correspond to points colors in **a** and **b**. **d**, **e** and **f** As **a**, **b** and **c** but varying ρ. **g**, **h** and **i** As **a**, **b** and **c** but varying θ.

the estimates obtained by model fitting.

The timing of the simulated epidemics is highly sensible to changes in the parameter values, while just slightly differences in the final epidemic size can be appreciated. In general, the final size varies from 38% to 41% whereas the epidemic peak week can change of more than 3 weeks.

Results are stable for large values of ρ, representing a fast imitation process, for small $x(0)$ values, i.e. if *altered* behavior is initially widespread in the population, and for a long lasting memory (Fig. S2). As varying $x(0)$ in a range $10^{-6} - 10^{-12}$ does not result in appreciable effects in model fit, we decide to fix $x(0) = 10^{-8}$, instead of searching for an optimum $x(0)$ value.

On the other hand, small variations of q — the size of the reduction on the force of infec-

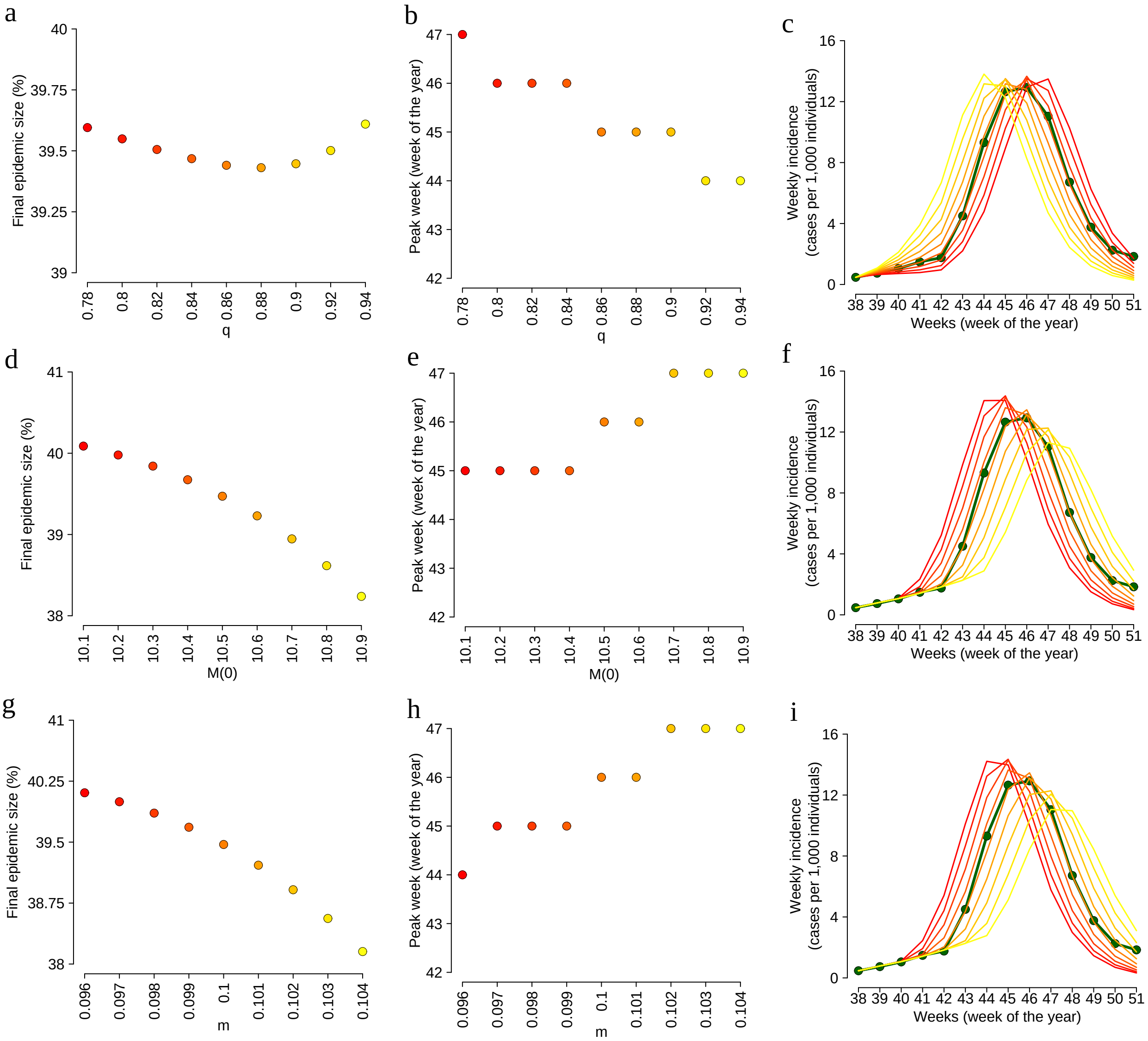

Figure S3: **a** Final epidemic size as simulated by the proposed model for different values of q. Other parameters as described in the main text (see Fig. 1). **b** As is **a** but for the peak week. **c** As is **a** but for the weekly incidence. Lines colors correspond to points colors in **a** and **b**. **d**, **e** and **f** As **a**, **b** and **c** but varying $M(0)$. **g**, **h** and **i** As **a**, **b** and **c** but varying m.

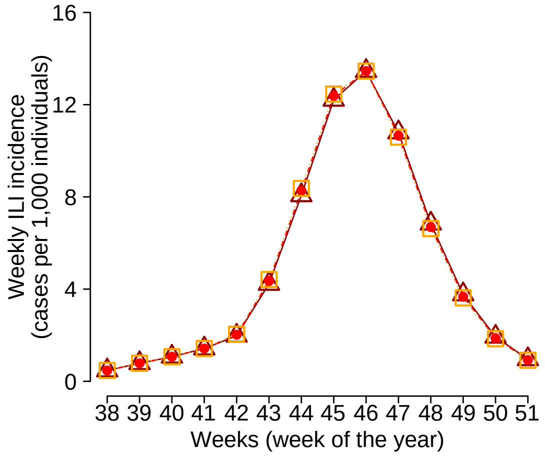

Figure S4: Weekly incidence as obtained by simulating the proposed model for three different sets of values of m and $M(0)$ (constrained to $m \cdot M(0) = 1.05$). Other parameters as described in the main text (see Fig. 1).

tion performed by individuals adopting the *altered* behavior — significantly influence the initial growth of the epidemic. This in turn strongly affects the peak week of the epidemic (Fig. S3 b, c). Specifically, for $q \in (0.78, 0.94)$, R_0^a lies in the range $1.1 - 1.4$. However, no significant differences can be appreciated in the final epidemic size (Fig. S3a). Indeed, a slower increase in the early phases of the epidemic would produce a lower number of cases, but would also accelerate the decrease of the perceived risk of infection, advancing the diffusion of the *normal* behavior in the population. In conclusion, the reduction of the force of infection in the early phases of an epidemic, due to an initial overestimation of the risk of infection, leads to a delay in the epidemic spread.

As for the risk threshold $1/m$ and the initial perceived prevalence $M(0)$, they both contribute to set the initial perceived risk of infection and they determine, through the memory mechanism, when it becomes more convenient to adopt the *normal* behavior. This in turn determines the period characterized by a growth rate of the epidemic lower than that expected in a population where no spontaneous behavioral changes occur. The larger is $M(0)$, the smaller is the final epidemic size and the more delayed is the epidemic peak (see Fig. S3 d, e and f). The same is observed for m (see Fig. S3 g, h and i). Specifically, variations of the order of 10% in $M(0)$ or in m result in absolute differences in the epidemic size of about 2% and, most remarkably, in variations of 3-4 weeks of the epidemic peak week.

Actually, the investigated situation is characterized by: *(i)* a risk of infection unable to sustain the *altered* behavior as a convenient choice; *(ii)* an initial overestimation of this risk of infection. In this specific case, the product $m \cdot M(0)$ is the crucial factor (rather than the values of the single parameters). In fact, as shown in Fig. S4, by varying values of m and $M(0)$ under the constraint that their product is kept constant, no appreciable differences in model trajectories can be detected.

In our investigation we have assumed a fixed generation time of 2.5 days, according to recent estimates [6, 7]. As shown in Fig. S5, predictions are not very sensitive to the length of the generation time (for $1/\gamma = 2$ and $1/\gamma = 3$).

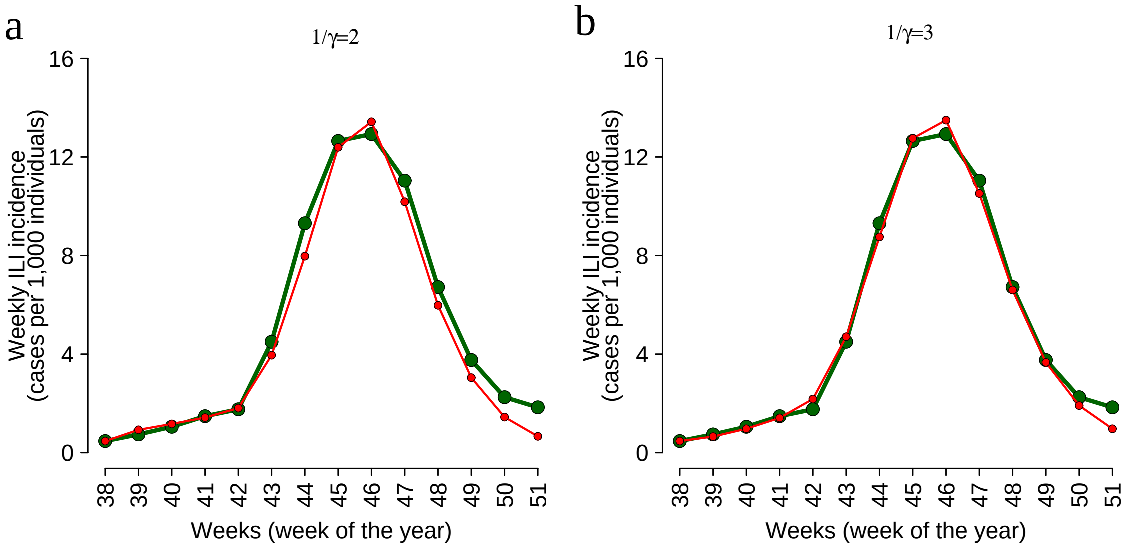

Figure S5: **a** Weekly ILI incidence as reported to the surveillance system (green) and weekly incidence simulated by the model by assuming $1/\gamma = 2$ days (red). Parameters values: $\beta = 0.719$, $q = 0.83$, $m = 0.1$, $\rho = 61.3$, $\mu = 0.005$, $M(0) = 10.5$, $x(0) = 10^{-8}$, $I(0) = 0.00121$ and $S(0) = 0.1$. The estimated basic reproductive number lies in the range $1.2 - 1.44$ and the reporting factor is 17.9%. **b** Weekly ILI incidence as reported to the surveillance system (green) and weekly incidence simulated by the model by assuming $1/\gamma = 3$ days (red). Parameters values: $\beta = 0.518$, $q = 0.84$, $m = 0.1$, $\rho = 64.9$, $\mu = 0.005$, $M(0) = 10.5$, $x(0) = 10^{-8}$, $I(0) = 0.00124$ and $S(0) = 0.1$. The estimated basic reproductive number lies in the range $1.31 - 1.55$ and the reporting factor is 15.4%.

In short, the model is able to account for the notable observed pattern, characterized by a sudden change in the slope of the incidence, if:

I an initial large diffusion of the *altered* behavior, due to a perceived risk of infection over the threshold, occurs;

II the *altered* behavior results as more convenient for a relevant period of time (e.g. thanks to a long-lasting memory);

III the imitation process is fast enough to allow a sudden change in the distribution of the behaviors adopted by the population, which in turn results in a sudden change of the growth of the epidemic.

In conclusion, $m \cdot M(0) > 1$ represents an overestimation of the initial risk of infection. The closer is the product $m \cdot M(0)$ to one and the larger is ρ, the smaller are the effects of the overestimation of the risk and thus the shorter is the period characterized by the diffusion of the *altered* behavior. As a consequence, as this period becomes smaller, the dynamics of the model becomes similar to the one predicted by a "simple" *SIR* model.

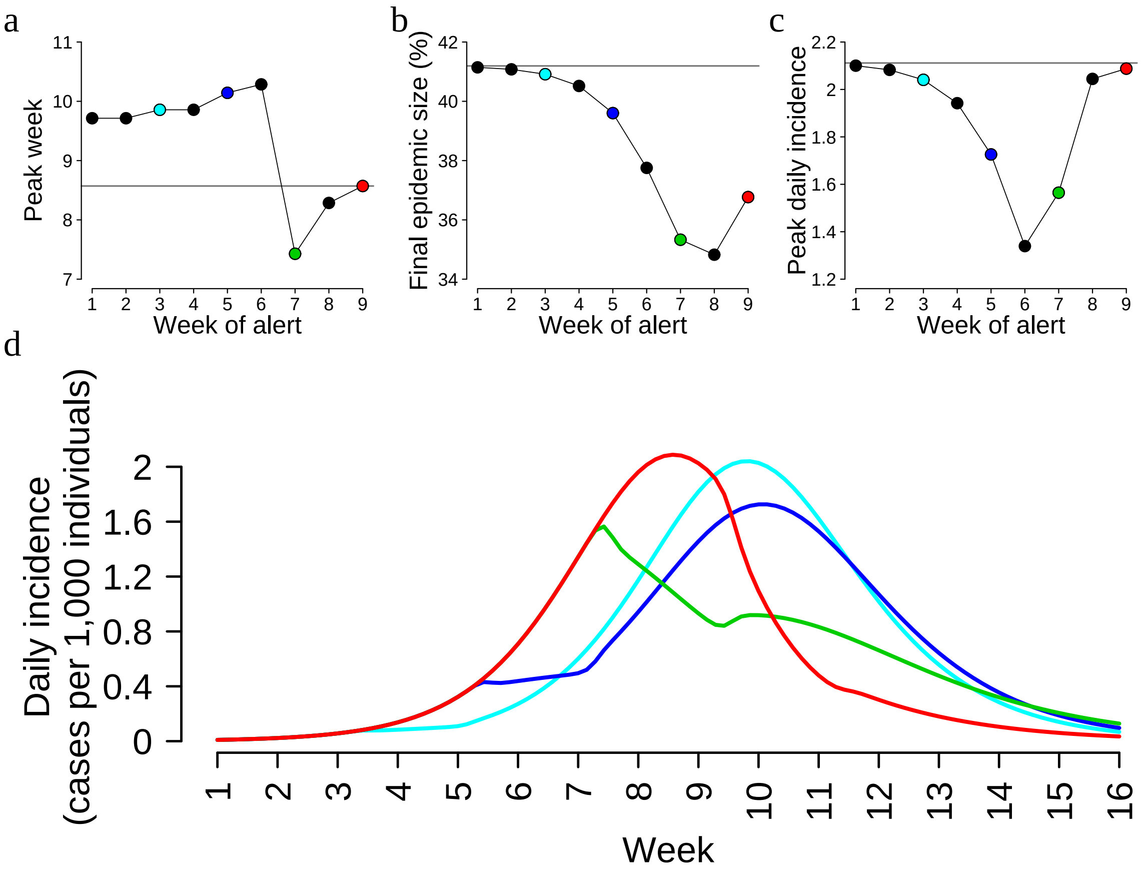

Figure S6: **a** Peak week as predicted by the model for different times of the alert. Parameters as in Fig. 1a of the main text, but for M, which is set to 10.5 at the time of the alert (same value as in Fig. 1a of the main text but different time). Horizontal black line represents the peak week as predicted by the "classical" SIR model. **b** Final epidemic size as predicted by the model for different times of the alert. Horizontal black line represents the final epidemic size as predicted by the "classical" SIR model. Parameters as in **a**. **c** Peak daily incidence (cases per 1,000 individuals) as predicted by the model for different times of the alert. Horizontal black line represents the peak daily incidence as predicted by the "classical" SIR model. Parameters as in **a**. **d** Daily incidence as predicted by the model for different times of the alert. Colors of the lines correspond to colors of the points in **a**, **b** and **c**.

4 Alert time

Our analysis has revealed that a central role for determining epidemic dynamics has been played by the initial concern about the spread of a new influenza pandemic. Here we investigate what would happen if the overestimation of risks occurs at different times (here considered as "alert" times), for example driven by different timing in the mass media information campaign. Specifically, as shown in Fig. S6, if the alert takes place during the early phases of the epidemic, no relevant effects are observed in terms of final epidemic size nor in the peak incidence, while

the diffusion of the virus can be slowed down allowing public health agencies to gain time to perform control strategies such as vaccination (which requires time for the preparation and the distribution of doses). Alerts performed during the course of the outbreak have limited effects in slowing the epidemic spread and thus they do not allow gaining time for the interventions. However, these alerts can result in lower peak incidence (and thus in a lower burden for health care centers) and in a relevant decline of the final epidemic size. Clearly, alerts performed at the end of the epidemic have no effects on the timing and on the peak incidence of the epidemics but may result only in a reduction of the final size as they can contribute to accelerate the decline of the epidemic.

5 Past influenza seasons

As shown in the main text, the proposed model perfectly fits the ILI incidence reported to the Italian surveillance system during the 2009 H1N1pdm influenza. Specifically, our analysis has shown that a self-protection behavior, spontaneously performed by the population in response to a high initial perceived risk of infection, represents a plausible explanation for the notable observed pattern. As discussed in the main text, this could have been induced by the mass media information campaign on the risks of an emerging influenza pandemic; such hypothesis is also supported by empirical evidence (such as the trend of antivirals drugs sales and the sporadic self-imposed school closures during October 2009). Our aim in this section is to investigate if behavioral changes spontaneously performed by the population have been a peculiarity of the 2009 pandemic or if (and possibly how) they could have played a central role during past influenza seasons.

Our analysis focuses on the last three influenza seasons (namely, the 2006-2007, the 2007-2008 and the 2008-2009) in Italy and, exactly as for the analysis shown in the main text, it is based on model fit to ILI incidence reported to the Italian surveillance system (data available at `http://www.iss.it/iflu/`).

We found that considering behavioral dynamics does not improve the accuracy of the fit with respect to a "simple" SIR model for the 2006-2007 and the 2008-2009 influenza seasons. Specifically, the best parameter sets estimated by using a least square fitting procedure for both 2006-2007 and 2008-2009 seasons force the system to a configuration characterized by the diffusion of a single strategy adopted by the population over the whole course of the epidemic (i.e., $x(t) = 0 \forall t$ or $x(t) = 1 \forall t$). Therefore, the proposed model and the "classical" SIR model coincide. Best model fits to the weekly ILI incidence in 2006-2007 and 2008-2009 influenza seasons are shown in Fig. S7 a and b.

As regards the 2007-2008 influenza season, we found that the proposed model, accounting for behavioral changes in the population, fits the weekly ILI incidence better than the "classical" SIR model (see Fig. S7 c and d). However, our parameters estimate suggests no initial overestimation of the perceived prevalence. The diffusion of an "altered" behavior could possibly have occurred during the most acute phase of the epidemic, close to the epidemic peak, i.e. when the risk of infection was "really" higher. Hence, even in this case, no overestimation of the perceived risk of infection at the beginning of the epidemic has been detected by our analysis, as opposed to that observed for the 2009-2010 season. Fig. S8 show the dynamics of the ILI incidence and of the

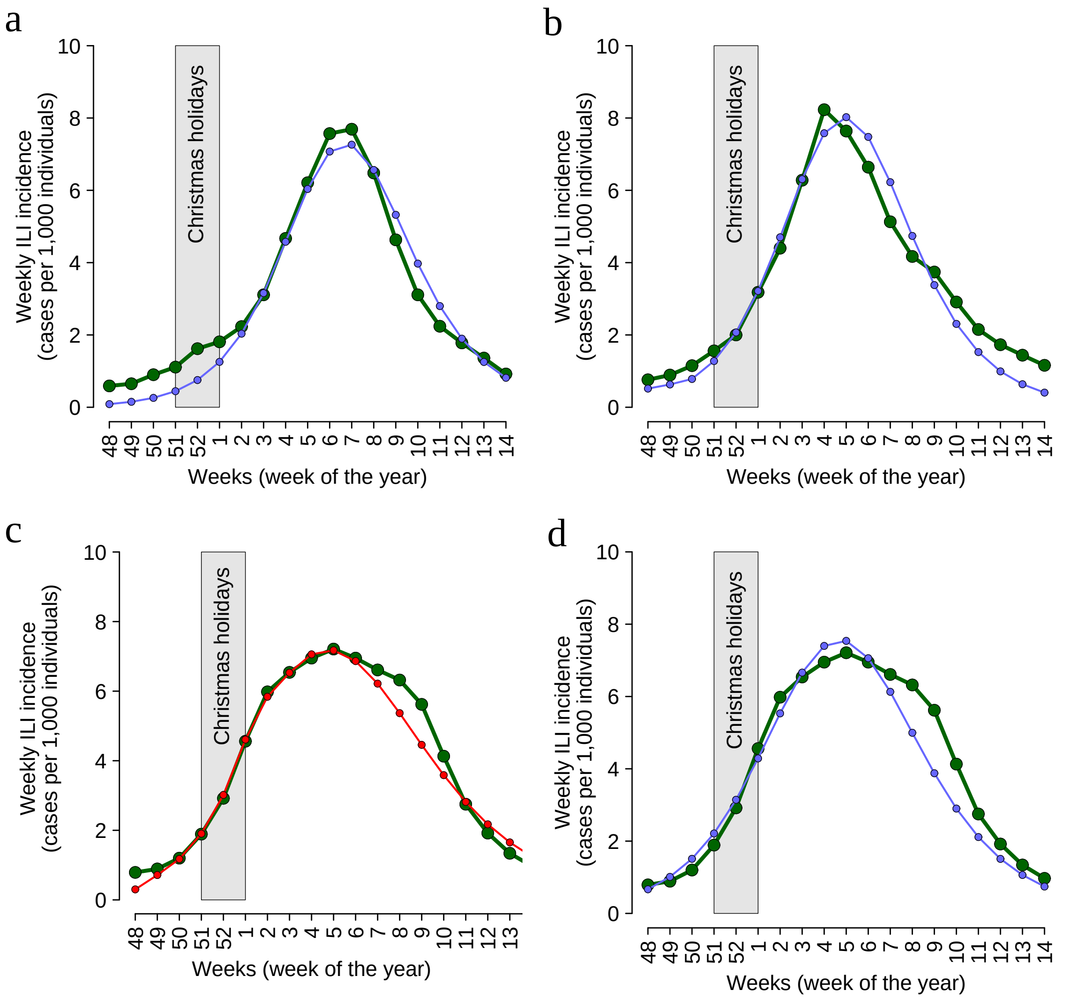

Figure S7: Analysis of the 2006-2007, the 2007-2008 and the 2008-2009 influenza seasons. **a** Weekly ILI incidence as reported to the Italian surveillance system (green) in the 2006-2007 season and weekly incidence as simulated by the "classical" SIR model (blue). Parameters values: $\beta = 0.49$, $\gamma = 0.26$, $I(0) = 0.001$. Since serological analysis on those influenza seasons are not available to us, the initial fraction of susceptible individuals in the population is $S(0) = 0.73$ as assumed in literature (e.g., in [8, 9]). The estimated effective reproductive number results to be 1.38 and the reporting factor is 15%. **b** Weekly ILI incidence as reported to the Italian surveillance system (green) in the 2008-2009 season and weekly incidence as simulated by the "classical" SIR model (blue). Parameters values: $\beta = 0.46$, $\gamma = 0.27$, $I(0) = 0.001$ and $S(0) = 0.73$. The estimated effective reproductive number results to be 1.24 and the reporting factor is 23%. **c** Weekly ILI incidence as reported to the Italian surveillance system (green) in the 2007-2008 season and weekly incidence as simulated by the proposed model (red). Parameters values: $\beta = 0.35$, $\gamma = 0.18$, $q = 0.89$, $m = 30$, $\rho = 400$, $\mu = 0.03$, $M(0) = 0$, $x(0) = 0.99$, $I(0) = 0.001$ and $S(0) = 0.73$. The estimated effective reproductive number lies in the range $1.26 - 1.42$ and the reporting factor is 26%. **d** Weekly ILI incidence as reported to the Italian surveillance system (green) in the 2007-2008 season and weekly incidence as simulated by the "classical" SIR model (blue). Parameters values: $\beta = 0.44$, $\gamma = 0.26$, $I(0) = 0.0001$ and $S(0) = 0.73$. The estimated effective reproductive number results to be 1.24 and the reporting factor is 27%.

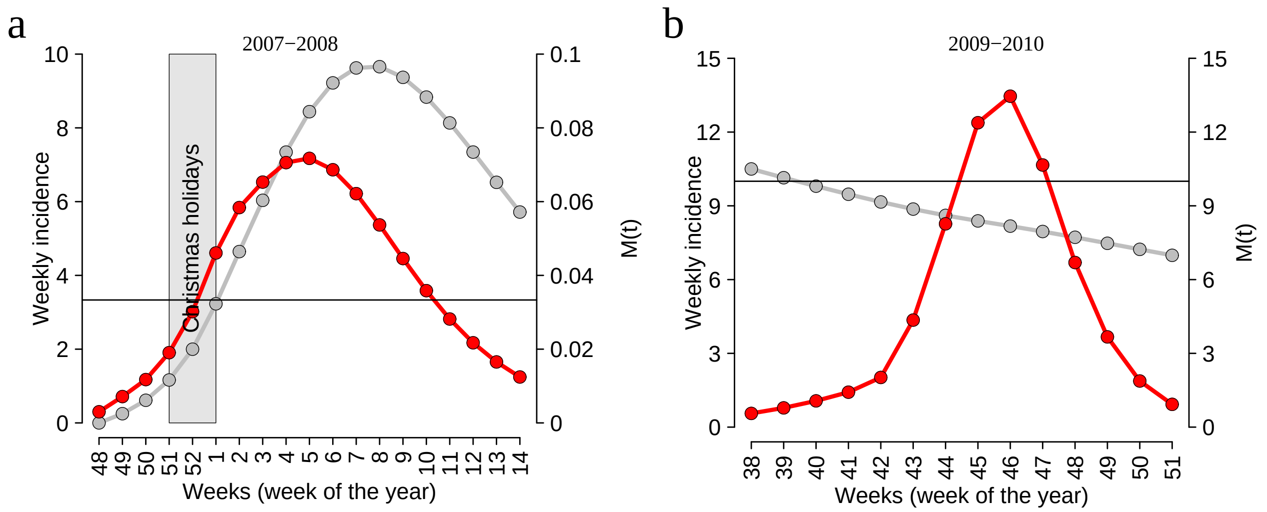

Figure S8: **a** Weekly incidence (cases per 1,000 individuals) as simulated by the proposed model (red) for the 2007-2008 season. Dynamics of the perceived prevalence (gray, scale on the right axis). Horizontal gray line represents the risk threshold. **b** As **a** but for the 2009-2010 pandemic season.

perceived prevalence, as obtained by fitting the 2009-2010 and the 2007-2008 influenza seasons.

In conclusion, this analysis suggests that behavioral changes would not have played a relevant role in past influenza seasons, at least in the early stages of the epidemic. The initial overestimation of the risk of infection seems to be a peculiarity of the 2009 pandemic.

6 Combining epidemiological and virological surveillance data

By combining virological [10] and epidemiological [11] surveillance data on the 2009–2010 season, we are able to estimate a theoretic lower bound for the weekly number of H1N1pdm infections. Specifically, we multiply the weekly ILI incidence to the number of laboratory confirmed cases divided by the number of tested specimens. Tested specimens have been sampled between the ILI cases identified by physicians participating to the national surveillance system.

As for the weekly ILI incidence (analyzed in the main text), this different dataset is characterized by two distinct exponential growth phases (especially appreciable in the log scale, see Fig. S9a). By fitting a simple SIR model and the proposed model to such data, we obtain substantially the same results discussed in the main text: the SIR model is unable to account for the initial phases of the epidemic, while the model including behavioral changes performs much better (see Fig. S9a, b).

We found that a simple SIR model with a time dependent reporting factor is able to capture the initial phase of pandemic only by considering extremely large values of the reporting factor (even above 100%, see Fig. S9c). Since this new dataset can be interpreted as a theoretic lower bound for the number of H1N1pdm infections, reporting factor values above 100% in principle

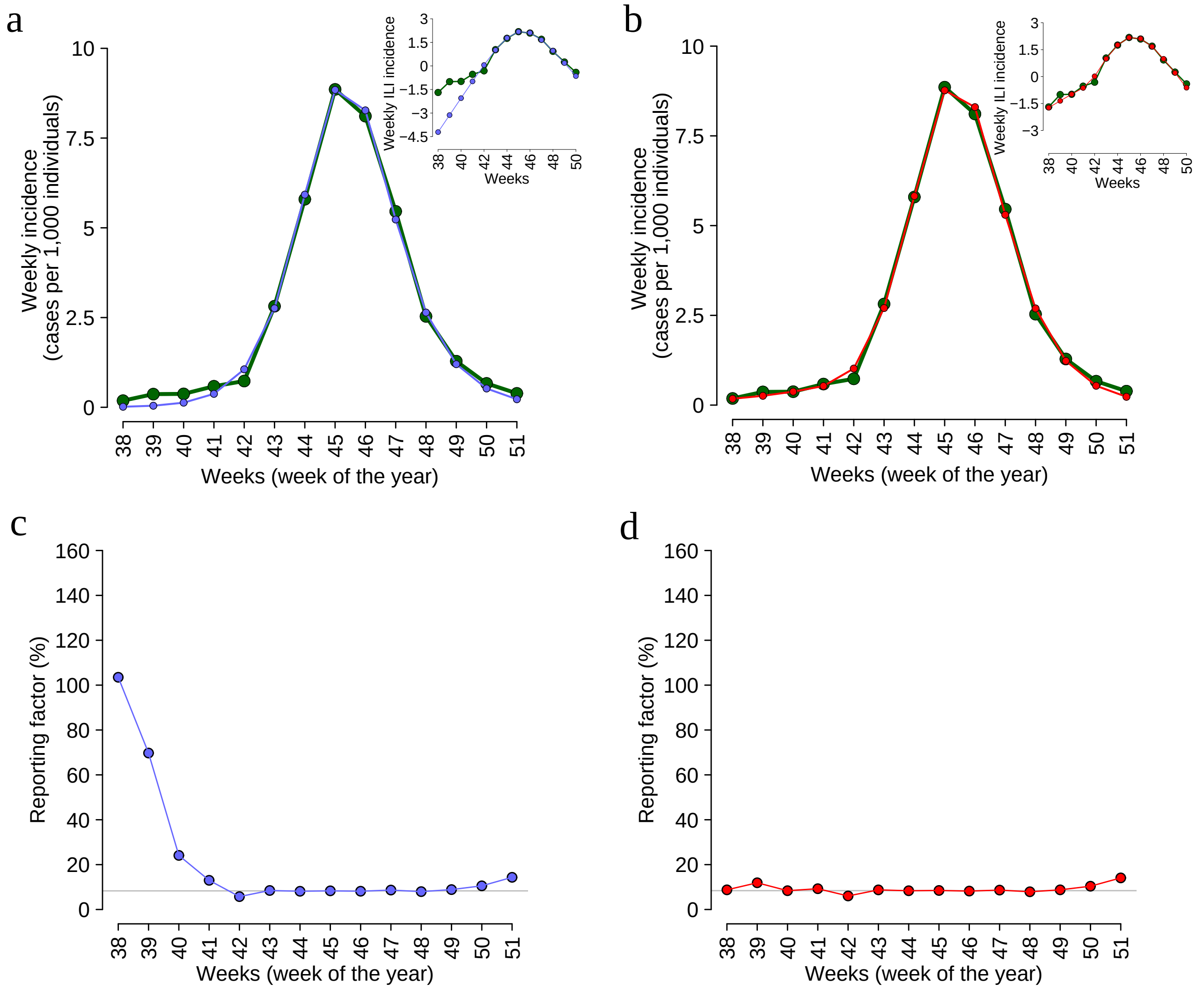

Figure S9: **a** Weekly incidence as obtained by combining epidemiological and virological surveillance data for the 2009 pandemic in Italy (green) and weekly incidence simulated by a "simple" SIR model with a constant reporting factor (blue). Sub–panel shows the same curves in a logarithmic scale. Parameter values used in the simulation are set as follows: the generation time $1/\gamma$ is assumed 2.5 days (in agreement with [6, 7]); $S(0) = 0.9$ (according to [12]); $I(0) = 0.000025$, $\beta = 0.62$, fitted. **b** Weekly incidence computed as in **a** (green) and simulated by the model accounting for behavioral changes (with a constant reporting factor, red). Sub–panel shows the same curves in a logarithmic scale. Parameter values used in the simulation are set as follows: $1/\gamma = 2.5$ days; $S(0) = 0.9$; $x(0) = 10^{-8}$, assumed; $M(0) = 10.17$, $I(0) = 0.00054$, $q = 0.815$, $m = 0.104$, $\rho = 70$, $\theta = 0.0046$, $\beta = 0.62$, fitted. **c** Weekly reporting factor estimates that enable the "simple" SIR model (parameters as in **a**) to exactly fit the influenza incidence (as computed in **a**). The horizontal gray line represents the average reporting factor as computed over the weeks 42–51. **d** As in **c** but for the model accounting for behavioral changes.

cannot be observable. On the contrary, the model considering behavioral changes does not require such large values for the reporting factor (see Fig. S9d). In conclusion, this analysis suggests that a variable reporting factor does not seem to be able to explain alone the observed pattern.

References

[1] Hofbauer J, Sigmund K (1998) Evolutionary Games and Population Dynamics. Cambridge University Press.

[2] Nowak MA, Sigmund K (2004) Evolutionary Dynamics of Biological Games. Science 303: 793-799.

[3] Bauch CT (2005) Imitation dynamics predict vaccinating behaviour. Proc R Soc B 272: 1669-1675.

[4] Poletti P, Caprile B, Ajelli M, Pugliese A, Merler S (2009) Spontaneous behavioural changes in response to epidemics. J Theor Biol 260: 31-40.

[5] d'Onofrio A, Manfredi P, Salinelli E (2007) Vaccinating behaviour, information, and the dynamics of SIR vaccine preventable diseases. Theor Popul Biol 71: 301-317.

[6] Fraser C, Donnelly CA, Cauchemez S, Hanage WP, Van Kerkhove MD, et al. (2009) Pandemic Potential of a Strain of Influenza A (H1N1) : Early Findings. Science 324: 1557-1561.

[7] Yang Y, Sugimoto JD, Halloran ME, Basta NE, Chao DL, et al. (2009) The transmissibility and control of pandemic influenza A (H1N1) virus. Science 326: 729-733.

[8] Longini IM Jr, Koopman JS, Haber M, Cotsonis GA (1988) Statistical inference for infectious diseases: risk-specific household and community transmission parameters. Am J Epidemiol 128: 845-859.

[9] Cauchemez S, Valleron AJ, Boelle PY, Flahault A, Ferguson NM (2008) Estimating the impact of school closure on influenza transmission from Sentinel data. Nature 452: 750-754.

[10] Italian Ministry of Health (2010). Sorveglianza virologica dell'influenza. (Available in Italian at `http://www.nuovainfluenza.salute.gov.it/nuovainfluenza/andamentoEpidemiaNuovaInfluenza.jsp?id=1223`).

[11] Istituto Superiore di Sanitá (2010). Stagione influenzale 2009/2010: Sorveglianza epidemiologica. (Available in Italian at `http://www.iss.it/iflu/risu/cont.php?id=141&lang=1&tipo=5`).

[12] Rizzo C, Rota MC, Bella A, Alfonsi V, Declich S, et al. (2010) Cross-reactive antibody responses to the 2009 A/H1N1v influenza virus in the Italian population in the pre-pandemic period. Vaccine 28: 3558-3562.
